# The Host-Microbe Interplay in Human Papillomavirus-Induced Carcinogenesis

**DOI:** 10.3390/microorganisms7070199

**Published:** 2019-07-13

**Authors:** Rei Wakabayashi, Yusuke Nakahama, Viet Nguyen, J. Luis Espinoza

**Affiliations:** 1 Faculty of Medicine, Kindai University, Osaka-Sayama, Osaka 589-8511, Japan; 2 Faculty of Medical technology, Hanoi Medical University, Ton That Tung 1, DongDa, Hanoi 100-000, Vietnam; 3 Department of Hematology and Rheumatology, Faculty of Medicine, Kindai University, Osaka-Sayama, Osaka 589-8551, Japan

**Keywords:** human papilloma virus, virus induced carcinogenesis, immunosurveillance, genetic variation, human microbiome

## Abstract

Every year nearly half a million new cases of cervix cancer are diagnosed worldwide, making this malignancy the fourth commonest cancer in women. In 2018, more than 270,000 women died of cervix cancer globally with 85% of them being from developing countries. The majority of these cancers are caused by the infection with carcinogenic strains of human papillomavirus (HPV), which is also causally implicated in the development of other malignancies, including cancer of the anus, penis cancer and head and neck cancer. HPV is by far the most common sexually transmitted infection worldwide, however, most infected people do not develop cancer and do not even have a persistent infection. The development of highly effective HPV vaccines against most common high-risk HPV strains is a great medical achievement of the 21st century that could prevent up to 90% of cervix cancers. In this article, we review the current understanding of the balanced virus-host interaction that can lead to either virus elimination or the establishment of persistent infection and ultimately malignant transformation. We also highlight the influence of certain factors inherent to the host, including the immune status, genetic variants and the coexistence of other microbe infections and microbiome composition in the dynamic of HPV infection induced carcinogenesis.

## 1. Introduction

Human papilloma virus (HPV) is a ubiquitous double stranded DNA virus that is associated with the development of various types of benign and malignant neoplasms in humans. More than 200 HPV types have been described so far and a number of them are transmitted through intimate skin-to-skin contact during sexual contact [1]. These viruses can also spread from mothers to babies during normal vaginal delivery, which might result in the development of recurrent respiratory papillomatosis (RRP) in the child, a rare condition where warts caused by HPV form in the respiratory tract [2,3]. Iatrogenic HPV infections have also been reported in the healthcare setting, especially via vaginal ultrasound transducers and other re-usable gynecological equipment [4].

Although there is a great variation in the prevalence of HPV infection worldwide, it is estimated that nearly all sexually active people around the world may get infected with some HPV type at some point of their lives, and thus HPV infection is considered the most common sexually transmitted infection in the world [1].

HPV infections have been causally associated with the development of various cancers, including, cancers of the cervix, vagina, and vulva in women; cancers of the penis in men; and cancers of the anus and of the head and neck in both women and men [5,6,7]. In addition, HPV infections may contribute to the risk of prostate cancer [8], and accumulating evidence appear to link HPV infection with the development of lung cancer, particularly in non-smokers [9,10]. Thus, based on their oncogenic potential, HPVs are divided into two categories: low-risk and high-risk HPVs, according to their ability to cause benign or malignant lesions over time in the infected people. Low-risk HPVs cause skin warts (skin papillomatosis) being HPV6 and HPV11 the strains implicated in the majority of cases (90%). High-risk HPVs refers to strains that have the potential to cause cancer in infected individuals and among them, HPV types 16/18 are responsibly for nearly 70% of HPV-related cancers. Other high risk HPV types include 33, 45, 31, 58, 52, 35, 59, 51, 56, 39 and 68 with variable frequency and world-wide distribution [1,5,7]. 

In nearly 50% of cases HPV infections are cleared by an appropriate immune response within six months of exposure and in the majority of cases (~90%), the virus become undetectable within 1–2 years [11]. Whereas this phenomenon is broadly described as “viral clearance,” accumulating evidence, especially from the observations that HPV reactivation is frequently observed in immunocompromised patients, suggest that indeed this may represent a state of immune control, where the virus is below detectable levels or viral latency, rather than viral clearance or eradication [1,11,12,13]. 

Several immunological factors, from both the innate and adaptive immune system play important roles in HPV recognition and elimination and in most cases these lines of defense are highly effective. However, HPVs have developed several mechanisms aimed to escape immune response, ranging from unspecific viral strategies, capable of downregulating various components of the innate immune system, to more sophisticated evasion strategies that turn ineffective the adaptive immune response against the virus, which ultimately results in persistent viral infections. In addition, once malignant transformation has started, the virus can hijack the immune system creating a chronic proinflammatory response favorable for cancer development and tumor progression. 

In parallel to the immune escape mechanisms of the virus, during the host/virus interaction, other important players are also involved. For example, host genetic variants in genes involved in human immune response may influence individual susceptibility to the long persistence of the virus and ultimately could influence HPV-induced cancers or tumor progression. Similarly, the presence of other microorganisms in the host, such as infection with other viruses, or alterations in the microbiota composition (dysbiosis) in the tissues infected with HPV, directly or indirectly may determine the virus infection outcomes. Consequently, HPV-induced carcinogenesis appears to be the result of a breakdown of the balance between the host and the virus, where the host’s defense mechanisms (especially the immune response) are overcome by the pathogenic factors of virus leading to a persistent infection that ultimately can cause cancer. 

In this article, we discuss current understanding on the virus/host interaction during HPV-induced carcinogenesis and highlight recent insights on the immune response against the virus, as well as on the possible implications of microbiome during such virus/human interplay that may be translated into novel preventive and therapeutic approaches against HPV-induced cancers. 

## 2. Fundamental Aspects of HPV Virology 

HPV is a nonenveloped double-stranded DNA virus with a circular genome (approximately 8000 base pairs) that can be physically divided into three regions: an early region of six early viral genes (E1, E2, E4, E5, E6, and E7) encoding non-structural proteins; a late region with two late genes (L1 and L2) encoding structural viral capsid proteins required for virion formation and virus spread [14,15]; and a long control region (LCR), which is a variable region that contains the early promoter and various transcriptional regulatory sites for both viral and cellular proteins [16]. 

Some HPV early gene products such as E1 and E2 are critical regulators of viral DNA transcription and replication and E2 especially contributes to maintaining the intracellular transport and nuclear accumulation of the DNA during infection [17]. Another early viral gene product E4, facilitates viral replication and is implicated in the disruption of cellular cytoskeleton to facilitate the exit of virions from infected keratinocytes [18,19]. E6 and E7 drive most of the cellular events leading to malignant transformation and thus they are considered the major oncogenic proteins of HPV. E6 product binds mainly to tumor suppressor protein p53 and induces cell cycle arrests in the S phase of the cell cycle and E7 binds and inactivates tumor suppressor pRB resulting in tumorigenesis [19]. E5 interacts with multiple host cell proteins and has been recently recognized as an oncoprotein whose carcinogenic properties include, stimulation of cell proliferation, inhibition of apoptosis induced by death receptors, and the modulation of genes implicated in cell adhesion and immune function [20]. 

HPV has evolved to synchronize its infectious cycle to the differentiation program of the target cells (keratinocytes) in the host. Once the virus gain entry into the epithelium via skin microlesions, it infects immature basal keratinocytes and very likely the stem cells [21,22] and while the viral episomes remain at a low copy number in basal cells, the amplification of the HPV genome and the subsequent production of infectious virions do not occur until the infected keratinocytes cells undergo terminal differentiation [19,22,23]. Thus, the time from infection to release of virus is approximately 3 weeks, which is equivalent to the time required for the basal keratinocytes to undergo complete differentiation and desquamation. As a result of the perfect synchronization of HPV with the epithelial differentiation program, cytolysis or cytopathic changes do not occur during the virus replication; and since the virus-infected keratinocytes naturally die after terminal differentiation on the surface of the skin, no major danger signals in the host cells are activated during the viral cycle and therefore it does not generate an inflammatory response [24].

Typically, during its viral cycle, HPV genome does not integrate into the host DNA, however, for yet undefined mechanisms, in some circumstances the HPV genome integrates randomly into the host DNA, in a process that includes the disruption of E2 expression, and since E2 is a negative transcriptional regulator of E6 and E7 viral oncogenes, when E2 is lost, E6 and E7 become actively expressed, promoting malignant transformation, which is further promoted by the ability of E6 and E7 oncoproteins to disrupt the tumor-suppressor genes p53 and pRB, respectively [21]. As a result, data from tumor models and cervical cancer tissues indicate that HPV-induced carcinomas exhibit three common features: integration of viral DNA (almost always) into the cellular genome; preservation and expression of E6 and E7 genes; and lost or lack of expression E2 and E4 genes [5,18]. 

Cervical cancer, the most frequent malignancy associated with HPV, typically develops from precancerous lesions over 10 to 20 years. About 90% of cervical cancer cases are squamous cell carcinomas, 10% are adenocarcinomas, and a small number are other types. The classification of cervical carcinoma precursor lesions has been modified several times over the years. For example, the world health organization classification system categorized cervical lesions into mild, moderate, or severe dysplasia or carcinoma in situ (CIS). Thereafter, the term cervical intraepithelial neoplasia (CIN) was developed to emphasize on the spectrum of abnormalities in these lesions in an attempt to standardize treatment. It classifies mild dysplasia as CIN1, moderate dysplasia as CIN2, and severe dysplasia and CIS as CIN3. More recently, CIN2 and CIN3 have been combined into CIN2/3. On the other hand, the Bethesda system is based on the Papanicolaou (Pap) test results and classifies lesions into Low-grade Squamous intraepithelial lesion (LSIL) and High-grade intraepithelial lesion (HSIL). Although they are results of different tests, an LSIL Pap may correspond to CIN1, and HSIL may correspond to CIN2 and CIN3. 

## 3. Immune Response to HPV Infection

Despite the high frequency of HPV infections, malignant transformation of infected tissues is not a common phenomenon suggesting that the immune system plays a central role in the control of these infections. This is substantiated by the increased frequency of HPV-associated cervical dysplasia observed in immunosuppressed patients and further supported by the effective immune response induced by anti-HPV vaccines. On the other hand, HPVs have evolved to infect the host and to some extend propagate almost inadvertently avoiding confrontation with the human immune system. As mentioned above, the synchronization of the viral cycle to keratinocyte differentiation process allows the virus to propagate without inducing a major inflammatory response and since HPV replication occurs in the epithelium, which is a poorly vascularized area, circulating immune cells cannot approach the virus easily. The lack of inflammatory cytokine release from infected keratinocytes limits the activation of resident skin Langerhans’s cells and dendritic cells, which are required for the effective induction of an adaptive immune response. 

In addition, accumulating evidence indicate that constitutively expressed cellular factors (restriction factors), including various intrinsic nuclear factors such as ND10 factor (sp100), IFIT1 (p56), IFI16, APOBEC, and others (reviewed in [25]) can directly restrict papillomavirus replication and transcription thus counteracting viral infection. Notably, HPVs have evolved evasion mechanisms that can target these restriction factors and silence their anti-viral effects.

### 3.1. HPV Interaction with Innate Immune System

Innate immunity is the first line of defense against pathogens, including HPVs, and is composed of nonspecific mechanisms including physical barriers, such as skin, soluble factors (cytokines and other inflammatory factors) and cellular components such as granulocytes (neutrophils, eosinophils, basophils) and non-granulocyte cells such as natural killer cells (NK cells), monocytes, macrophages and dendritic cells, which constitute an efficient bridge that connects the innate immune response with the adaptive immune system. 

HPVs have developed strategies that efficiently evade or inhibit the innate immune response. For example, E7 oncogene, and to a lesser extend E6, reduce NFκB activation [26], which severely impairs immune response, since NFκB activation is critical for the production of proinflammatory cytokines like IL-1, IL-6 tumor necrosis factor alpha (TNF-α), interferons (IFN-α and IFN-β) that activate immune response [27]. High risk HPV16 and 18 are able to inhibit the transcription of proinflammatory chemokines and cytokines, such as CCL5 and IL-1β [28] and in a similar fashion, the IFN inducible antiviral genes IFIT1 and MX1, as well as proapoptotic genes (TRAIL and XAF1) are also inhibited by HPV16, 18, and 31 [29]. 

Most cells of the innate immune system express pathogen recognition receptors (PRRs) such as cytosolic retinoic acid-inducible gene I (RIG-I)–like helicases for the recognition of viral RNA and several members of the toll-like receptors (TLR) family that also play a crucial role in innate immunity. Within the TLR family, TLR9 is implicated in the recognition of double-stranded DNA CpG motifs present in the genome of viruses like HPVs. TLR9 activates cellular signals that lead to the production of type I IFN and proinflammatory cytokines during viral infections [30]. However, infection of human primary keratinocytes with HPV16 inhibits TLR9 expression, where E6 and E7 oncoproteins directly down-regulate TLR9 at transcriptional level [31]. This is consistent with the drastic downregulation of TLR9 expression observed in tissue specimens derived from women with cervical lesions associated with HPV16 infections in comparison with normal tissues or with specimens derived from women infected with low-risk HPVs [31,32].

Professional antigen presenting cells (APCs), such as dendritic cells (DC), macrophages, and subsets of B cells are immune cells that constitutively express Major Histocompatibility Complex class II (MHC class II) molecules on their surface and specialize in presenting antigens to T-cells, thus playing a critical role in the activation of the T cell-mediated immunity. Conversely, non-professional APCs constitute a variety of immune and non-immune cells including fibroblasts, keratinocytes thymic epithelial cells, thyroid epithelial cells, glial cells, pancreatic beta cells, and vascular endothelial cells, which do not constitutively express MHC class II proteins, can express these molecules upon cytokine stimulation, especially IFN-γ, and may thus interact with and present antigens to naïve T cells [33]. DCs play a pivotal roles linking the innate and the adaptive immune systems and in the context of HPV infection, skin DCs cells in the context of HPV infection, DCs are the only professional APCs found in the skin capable of efficiently cross-presenting HPV-associated antigens and transport processed antigens to the draining lymph nodes to activate proliferation of cytotoxic CD8+ T cells [34], thus DCs play critical roles in priming virus-specific T cells and are essential generating antitumor cytotoxic CD8+ T cells. However, HPVs impair the migration, recruitment and localization of dendritic cells to HPV-infected epidermis [35] and inhibit the maturation and function of skin DCs, thus limiting their capacity to stimulate cytotoxic CD8+ T cells [35,36]. Therefore, given the central role of these cells in the generation of an effective antiviral immune response, impairing DC function constitutes one of the most important immune evasion strategies of HPVs leading to persistent infection and malignant transformation.

Keratinocytes, the predominant cell type in the epidermis, play a critical role during HPV infection not only because they are the cells were HPVs proliferate but also because they have innate immune-like functions. For example, keratinocytes express various TLRs, including TLR9 and upon stimulation they secrete various cytokines like TNF-α, IL-8, CCL2, CCL20, CXCL9 and type 1 IFNs [24]. As mentioned above, keratinocytes also act as non-professional APCs contributing to the activation of the adaptive immune system [37]. Notably, HPVs inhibit the production of proinflammatory cytokines from infected keratinocytes via E6 and E7 mediated inactivation of NF-κB signals [24,37] and by inducing the downregulation of TLR9 of the surface of these cells [38]. Further, CCL20 secreted by keratinocytes is required for the migration of immature Langerhans’s cells to the epidermis [39], where the adherence of keratinocytes to Langerhans’s cells, via E-cadherin, is required for Langerhans’s cells differentiation [40]. Experimental data showed that E7 oncoviral protein inhibits CCL20 expression in human primary keratinocytes by preventing binding of the cellular transcription factor CCAAT/enhancer binding protein (C/EBP) to the CCL20 promoter [41] and also via downregulation of NF-κB signaling [42], which ultimately results in impaired Langerhans’s cells migration to the epidermis.

Macrophages contribute to the clearance of HPV infections by eliminating HPV infected cells [43] and by secreting proinflammatory cytokines such as TNF-α and IL-17A that promote the infiltration of other immune cells to the site of infection [44], however HPVs impair macrophage translocation during acute infection by interfering with macrophage chemotaxis. In particular, oncoproteins E6 of HPV inhibit the release of monocyte chemotactic protein MCP-1 from infected keratinocytes [45]. In the same way, E6 and E7 oncoproteins of HPV16 downregulate the secretion of macrophage inflammatory protein 1α (MIP1α) from infected keratinocytes [42]. It must be noted however that once malignant transformation induced by HPV has been established, tumor-infiltrating macrophages (TIM), indeed promote tumor progression [46].

NKG2D-Ls are molecules expressed on cells exposed to stress stimuli such as virus infection, radiation or malignant transformation and these ligands can be recognized and killed by immune cells expressing the immunoreceptor NKG2D, including NK cells, γδT cells, and other T cells subsets. Interestingly, NKG2D-Ls (ULBP1, MICA/B and ULBP2) have been detected in cervical cancer cell lines [47,48] and their expression in tumor tissues is an indicator of good prognosis in patients with cervical cancer, suggesting that ligand-expressing cells can be targeted by NKG2D receptor-expressing immune cells in vivo [49].

E7 oncoprotein directly interacts and blocks the major histocompatibility complex class I (MHC-I) heavy chain promoter leading to reduced MHC-I expression on infected cells [50]. Similarly, E5 oncoprotein contributes to MHC-I downregulation on cell surface by sequestering HLA-C and HLA-E in intracellular compartments [20,51]. Although HPV infected cells lacking HLA class I expression on their surface escape cytotoxic T-cells attack, as HLA class I recognition is required to initiate cytotoxicity mediated by T-cells, they can be targeted by NK cells, which are educated to eliminate HLA class I deficient cells [52], however, malignant cells can evade NK cells cytotoxicity by multiple mechanisms. For example, HPV-induced cancers can shed NKG2D-Ls in an attempt to evade NKG2D mediated killing by immune cells and indeed elevated serum level of MICA protein have been reported in patients with cervical cancer and precursor lesions, which is associated with significant reduction of NKG2D receptor expression on NK and T cells [53].

Indoleamine 2,3-dioxygenase 1 (IDO1) is a naturally immunosuppressive molecule that regulates immune function by controlling tryptophan levels. In normal conditions, IDO1 controls excessive immune activation preventing autoimmunity, however, tumor cells upregulate IDO1 activity by depleting tryptophan in the tumor microenvironment [54]. Dermal dendritic cells from grafted skin expressing HPV16 E7 oncoprotein express high levels of IDO. Similarly, high expression of IDO1 and IFN-γ were detected in the cervical epithelium of patients with HPV-associated CIN 2/3 [55]. Notably, IDO expression substantially impairs NK cell cytotoxicity and correlates with the escape of tumor cells from immune surveillance [15].

Recently, using patient tissue specimens and cultured keratinocytes, Cicchini and colleagues found that oncoprotein E7 induces hypermethylation of CXCL14 promoter leading to the downregulation of the chemokine CXCL14. In murine models of head/neck and cervical cancers, restoring Cxcl14 expression in HPV-positive cancer cells dramatically increased NK and T cells in the tumor-draining lymph nodes, leading to potent anti-tumor responses in vivo [56]. CXCL14 is a chemokine widely expressed in normal tissues that is secreted by endothelial cells, keratinocytes, fibroblasts and certain immune cells and appears to be lost or significantly down-regulated in various malignancies [57]. In addition to promote NK cells migration, CXCL14 can attract immature dendritic cells, monocytes, neutrophils and human uterine NKs [57]. Interestingly, CXCL14 is constitutively expressed in the epidermis of healthy skin [58] and is involved in the recruitment of CD14+ DC precursors and ultimately influences their differentiation into Langerhans cells [59]. The fact that CXCL14-producing fibroblasts co-localize with macrophages suggests a role for CXCL14 in macrophage development [60]. Thus, given the important role of CXCL14 in the activation of cells implicated in the immune response against HPV, it not surprising that the virus has evolved a mechanism to deplete this chemokine as an immune escape strategy.

Oncoproteins E6 and E7 of high risk HPVs also impair immune response by promoting the production of transforming growth factor β (TGFβ) by increasing TGF-*β*_1_ promoter activity [61]. As a result, increased TGFβ expression has been observed in HPV+ tumors and TGFβ may induce local immune suppression that impairs immune surveillance and therefore may contribute to increased susceptibility to malignant transformation [62].

### 3.2. HPV Interaction with Adaptive Immunity

Cell mediated immunity mediated by both, T-helper cells (Th cells, CD+) and cytotoxic T-cells (CTL, CD8+), plays a pivotal role in the elimination of HPV infections and in the regression of HPV-induced premalignant lesions [63]. For example, in patients with HPV-positive cancers, increased infiltration of CD3+CD8+ lymphocytes in stromal areas tumor tissues [64], and in intraepithelial areas [63], is associated with better survival. Higher numbers of B cells and CD8+ T cells infiltrating tumors were associated with better clinical outcomes in HPV-positive head and neck squamous cell carcinoma (HNSCC) patients [65] and these observations were further confirmed in a large meta-analysis showing improved overall survival in HPV-driven OPSCCs with increased levels of tumor-infiltrating lymphocytes [66].

Functionally, Th cells are phenotypically divided into Th1 and Th2 subsets. Th1 cells primarily produce proinflammatory cytokines (IFNγ, IL-1β, IL-18 and IL-2) and generate responses against intracellular pathogens such as bacteria and viruses. Conversely, Th2 cells produce IL-4, IL-5, IL-6, IL-10, and IL-13 and generate immune responses against extracellular pathogens. Interestingly, HR-HPVs have the ability to shift the immune response from Th1 to Th2 during early HPV-induced lesions, which harness antiviral responses, thus promoting disease progression [67].

The immunoregulatory molecules programmed death-1 (PD-1, CD279) and its ligand PD-L1 (CD274) have been linked to HPV carcinogenesis. Ligation of PD-1 (which is expressed on lymphocytes, particularly T cells) by its ligand PD-L1 (which can be expressed by malignant cells, as well as T cells, NK cells, macrophages, myeloid DCs, B cells, epithelial cells, and vascular endothelial cells) induces an inhibitory signal in activated T cells leading to T-cell apoptosis, anergy and functional exhaustion. Increased PD-1 and PD-L1 expression has been found on HR-HPV infected cervical tissues and its expression was correlated with increasing CIN grade [68,69]. In addition, cervical cancer tissues from patients previously treated with chemotherapy showed increased expression of PD-L1 in malignant cells and overexpression of PD-1 and CD8+ tumor infiltrating lymphocytes [70]. These observations point to a key role of the PD1/PD-L1 axis in immune escape of HPV-induced cancer.

Moreover, in preclinical studies, microparticles shed from HPV16 E7-expressing keratinocytes suppressed the cytotoxicity of CD8+ T cells, which was attributed to the downregulation of CD40 and IL-12 in Langerhans cells [71], indicating that those E7-microparticles from HPV-infected cells could suppress the T cell response thus contributing to persistence of HPV infection and cancer.

Natural HPV infection generates a specific humoral immune response against various viral antigens, as demonstrated by a high prevalence of naturally acquired antibodies to specific HPV types in individuals without cancer, in both sexes and all age groups [72]. These antibodies, which mainly recognize the major capsid protein L1, are neutralizing and may be protective against disease but develop slowly (average time of 8–9 months after the first detection of HPV16 DNA) and are detected only in a fraction of infected individuals [73]. Conversely, anti-L1 neutralizing antibodies elicited by virus-like particles (VLP) of prophylactic vaccines are more abundant and remain in the serum at high levels and for long-term, thus indicating that an effective humoral immunity is critical for the prevention of HPV infections [74]. In this regard, robust and persistent antibodies to HPV6, 11, and 16 have been even found in HIV-infected children with reduced CD4+T lymphocytes [75], thus substantiating the high immunogenicity of anti-HPV vaccines.

Humoral immunity against HPVs may predict the risk of malignant transformation in HPV infected individuals, although this may depend on the type of cancer. For example, high levels of anti-HPV16 E6 antibodies were detectable in most patients with oropharyngeal carcinomas (OPCs) more than 10 years before diagnosis [76] and in one third individuals before they developed anal cancer. Conversely, these antibodies were detected in only 0.6% of controls and in 1.5%, 3.3%, and 8.3% of patients with cancers of the vulva, cervix, and penis, respectively [77].

Regulatory T cells (Treg) are a subset of T cells expressing the transcription factor Foxp3 that are required for the induction of immune tolerance and auto-immunity prevention. Given the immunosuppressive functions of Treg cells, one may expect that HPVs promote the expansion of these cells, as a viral strategy to evade immune surveillance. This assumption is supported by the observations that HPV-induced lesions have elevated numbers of infiltrating FOXP3+ Tregs cells and Foxp3 expression is associated with increased odds of progression to high-grade lesions [78,79]. Further, preclinical studies have shown that FOXP3+ Tregs cells inactivation or depletion results in induce strong intratumoral invasion of CD8+ T cells and complete tumor eradication [80]. Various studies have reported that an increased FoxP3+ Tregs infiltration in tumor specimens correlates with worse prognosis in cervical cancer [80,81]. Similarly, patients with HNSCC have an increased circulating number of CD45RA-Foxp3high Tregs, which correlates with tumor stage and nodal status [82].

In tissue specimens from laryngeal squamous cell carcinoma (LSCC), accumulation of activated Treg cells and M2 macrophages (a subset of macrophages with immunosuppressive activities that are associated with increased tumor progression) was associated with adverse prognosis and a positive-feedback loop between activated Treg cells and M2 macrophages is crucial to the establishment of the immunosuppressive tumor microenvironment that is typically observed in these malignancies [79].

Interestingly, a recent study showed that in OPSCC specimens, tumor infiltration by Treg cells expressing Tbet+ (Foxp3+ Tbet+ Tregs) was strongly correlated with a concomitant tumor-specific and type 1-oriented intratumoral T cell infiltrate where an increased infiltration Tbet+ Tregs was associated with improved clinical outcomes [83].

**Figure 1:**
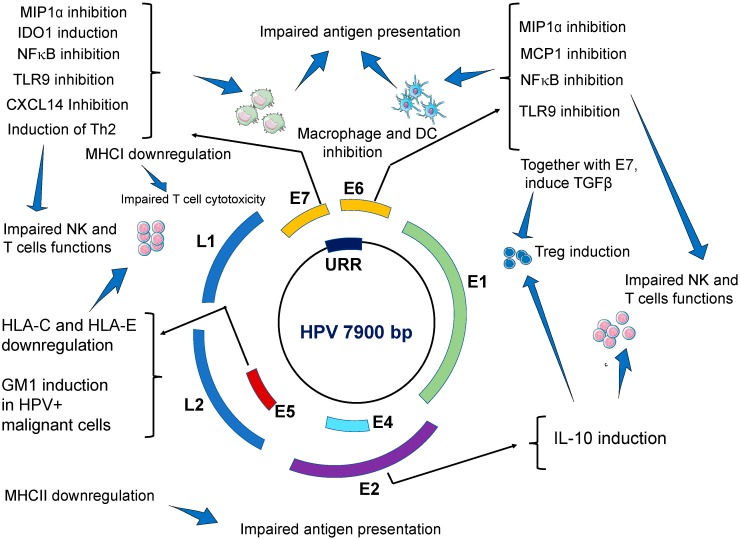
Viral gene products of HPVs and their effects on host immune response. Several HPV gene products, especially E2, E5, E6, and E7 exert a variety of inhibitory effects on several components of the host immune system which result in impaired antigen presentation, weakened natural killer (NK) cells and Treg (T) cells cytotoxic activities, and deficient immunosurveillance, which promotes the establishing a persistent infection that can ultimately result in malignant transformation.

In summary, important mechanisms implicated in the evasion of immune responses by HPVs include perturbing antiviral activities of innate immune system, hampering antigen processing and presentation, inducing a shifted Th1 to Th2 immune response, promoting immune anergy via the recruitment of regulatory T cells and likely by hijacking regulatory mechanisms of T cells apoptosis (Table 1 and Figure 1).

## 4. HPV Interactions with the Host Genetics

Single nucleotide polymorphisms (SNPs) constitute the most common form of genetic variation in the human genome and depending on their location within a given gene, SNPs can alter gene expression or protein function. Numerous SNPs are associated with disease predisposition and many have been reported to associate with individual’s susceptibility to cancer, including HPV-induced cancers. 

Human leukocyte antigen (HLA) genes, which are highly polymorphic are among the most studied factors since certain HLA variants are consistently associated with numerous inflammatory disorders and cancer. For example, women harboring the DRB1*1301 variant appear to have lower risk of developing cervical cancer [91,92]. Similarly, individuals with the DRB1*1302 allele are less likely to progress from CIN1 to CIN 2/3 [93].

Genetic variants in non-HLA genes have been also reported in association with HPV-related cancers. For example, rs3087404 and rs2029167 SNPs in *SMUG1* gene were associated increased susceptibility of CIN III and CSCCs [94].

The immunosuppressive cytokine TGFβ1 impairs NK cells cytotoxicity by promoting the downregulation of NK cells activating receptors including NKp30 and NKG2D [95] and high serum levels of TGFβ1 have been identified in cancer patients, including in HPV-related malignancies [62,96]. The SNP rs1982073 in *TGF-β1* has been reported to confer individual susceptibility to HPV+ OPC [97]. Importantly, the genotype CC of variant rs1982073 correlates with increased levels of TGF-β1 in serum as compared to the TT genotype; and this correlation was also seen among HPV16 positive patients [98]. Although the molecular mechanisms of TGF-β1 expression by rs1982073 have not yet been elucidated, these results might provide preliminary evidence of biological plausibility for the observed association in those studies.

SNPs in various genes encoding immunoreceptors have been also associated with increased risk to HPV-induced cancers. Our group reported an association between the variant LNK, determined by the SNP rs1049174 in the 3′UTR region of *KLRK1* gene (which encodes NKG2D receptor), with increased risk of HPV-induced cancers, including cervical cancer, anal cancer and vaginal cancer [48]. Mechanistic studies showed that rs1049174 genetic variant regulates NKG2D receptor expression and NK cells cytotoxicity. NK cells from individuals with the HNK genotype express higher levels of NKG2D receptor on their surface and have higher cytotoxic activity compared with NK cells from individuals harboring the LNK genotype. As a result, individuals with the HNK genotype have reduced risk of various malignancies [99,100,101]. Experimental evidence demonstrated that the NKG2D gene with the LNK allele is more sensitive than the HNK allele to degradation by the micro-RNA miR1245 due to an increased affinity and thereby this microRNA more efficiently downregulate NKG2D expression in NK cells with the LNK genotype [102]. Notably, NK cells from individuals with the LNK genotype are more susceptible to the down regulatory effects of TGFβ1 on NKG2D receptor expression [48]. The fact that NKG2D receptor is significantly downregulated in patients with HPV-related cancer, coupled with experimental evidence linking the oncoproteins E6 and E7 of HPV in the induction of TGFβ1 in host cells [61] suggest that HPV-induced cancers exploit TGFβ1 signal to escape immune responses mediated by NKG2D receptor.

Killer-cell immunoglobulin-like receptors (KIRs), constitute a large family of transmembrane proteins expressed on the surface of NK cells and a minor subset of T cells that interact with specific allelic variants of MHC class I molecules expressed on all nucleated cell types, thus regulating the cytotoxic functions of NK and T cells. Most KIRs are inhibitory, meaning that after recognizing their corresponding MHC molecules, they suppress the killing activity of effector cells (NK or T cells), however, a limited number of KIRs are indeed activating, implying that their recognition of MHC molecules activates the cytotoxic activity of effector cells [103]. Interestingly, patients lacking activating *KIR* genes *3DS1* and *2DS1* are more likely to develop a more severe form of RRP (caused by HPV-6/11) than those harboring these receptors [2], pointing out that NK cells are necessary to trigger an effective immune response against HPV-infected targets. Since KIR genes are highly polymorphic, it is likely that SNPs in select *KIR* genes may be implicated in the development of HPV-induced cancers or in the risk to develop persistent infections and considering the fact that various KIR inhibitory antibodies are been tested in the clinical setting [104], further studies that include a large number of individuals in different populations are needed to identify potential associations between KIR genes with HPV infection and its clinical relevance are warranted.

It is plausible that certain generic variants are relevant for some populations while have little or no impact in other populations. A recent meta-analysis that included 98 publications reporting genetic association studies of SNPs with risk to cervical cancer in Indian women revealed that only rs1048943 of *CYP1A1* to be significantly associated with cervical cancer [105].

In summary several studies have shown that genetic variants in certain genes, especially functional SNPs capable of affecting gene expression or altering the function of the encoded protein, may contribute to HPV-induced carcinogenesis, however, as occurs with many gene association studies, some findings are still inconclusive with some studies report contradictory associations across different populations and even in subjects of the same ethnicity (Table S1). Given the complex interaction between HPV and the infected host, it is unlikely that genetic factors constitute strong determinants of disease susceptibility in HPV-carcinogenesis, however genetic variants in critical components of cancer immunosurveillance may play and important role in individual predisposition to these malignancies.

## 5. HPV Coinfection with Other Microorganisms

Immunodeficiency associated with HIV infection is associated with impaired immune response of HPV infections. As a result, HIV+ women have increased risks of persistent HPV infection and higher risk to develop pre-malignant lesions and HPV-induced cancers and unfortunately, highly active antiretroviral therapy (HAART) appears to have limited ability to clear HPV infection and induce regression of premalignant lesions [106,107].

In a large study that included a total of 2791 HIV-infected and 975 HIV-uninfected women, followed up with Pap tests and colposcopy, women with HIV that were positive for HPV16 had 13-fold (*p* = 001) greater CIN-3+ risk than HPV-16 negative women. Similarly, HIV-infected women with LSIL had 9-fold (*p* < 0001) greater risk, indicating that HIV-infected women with a normal Pap result who test HPV16 positive have high pre-cancer risk [108]. However, in a multicenter prospective study conducted in the USA, (420 HIV-infected women and 279 HIV-uninfected women with normal cervical cytology) the 5-year cumulative incidence of HSIL+ and CIN-2+ was similar in HIV-infected women and HIV-uninfected women [107].

Various studies support the conclusion that HPV affects the local microbiome. For example, increased microbial diversity with a decreased frequency of *Lactobacillus* spp. was observed in HPV-positive women in comparison with HPV-negative women and fusobacteria, especially *Sneathia* spp., were proposed as a possible microbiome marker of HPV infection [109]. In another study that investigated the microbiome in cervical cytobrush samples from 144 women, HIV-positive patients were more likely to be positive for one or more HPVs and had greater bacterial richness than HIV-negative patients. HIV-negative samples infected with HPV had a 10-fold increase in *Bacteroidetes* and *fusobacteria* and showed a decrease in *Actinobacteria*. Conversely, among HIV-positive patients, *Mycoplasma* was more abundant and was the main bacteria differentiating the cervical microbiome of HIV-positive and HIV-negative individuals and correlated with the presence of HPV-related cervical lesions [110], indicating that the bacterial microbiota can influence the outcome of HPV infection. In line with these observations, a meta-analysis that included a total of 101,049 women to assess the impact of vaginal dysbiosis, HPV infections and progression to cervical pre-malignancy, identified a causal link between vaginal dysbiosis with HPV persistence, cervicovaginal dysplasia and cervical cancer development [111].

Preclinical studies have documented that changes in microbiota composition can alter local innate immunity. For example, infection with *Atopobium vaginae*, a prevalent microorganism in bacterial vaginosis, induced changes consistent with a significant disruption of immune barrier properties, including increased expression of membrane-associated mucins and a robust induction of proinflammatory cytokines (CCL20, IL-1β, IL-6, IL-8 and TNF-α) [112]. These findings are consistent with the notion that bacterial vaginosis may increase susceptibility to HPV infection. Since the primary defense mechanisms of the cervicovaginal mucosa are mucosal or commensal derived peptides con antimicrobial properties, as well as the maintenance of a low pH (<4.5), certain members of the microbiome community in the cervicovaginal niche may play a pivotal role in the prevention of colonization with deleterious microbes and therefore perturbations in this balance may lead to biochemical changes that result in alterations of the vaginal mucosa and cervical epithelium [113,114].This notion is consistent with the observations that an increased abundance of *Lactobacillus crispatus* inversely associated with symptomatic viral infections, including HIV, HPV, or herpesvirus infections [115,116] and with the observations that the loss of *Lactobacillus* colonization promotes the growing of anaerobic bacterial species, thus altering the microbial diversity [117].

Changes in the oropharyngeal microbiome composition may be also associated with HPV-induced cancers like OCSCC/OPSCC, as a recent study found integration hotspots for HPV16 along with other identified integration sites for a number of viruses, including the JC polyomavirus, as well as other pathogenic bacteria (*Mycobacterium, aeromonas, Sphingomonas, Bordetella and E.coli)* fungi and parasites in these OCSCC samples [118].

It is plausible that HPV can directly or indirectly induce changes in the infected tissues that affect the local microbiota composition and vice versa, dysbiosis of the local microbiota may in turn create conditions favorable for the colonization and the development of persistent infections with HPV or ultimately promote the development of precancerous lesions in a similar fashion as dysbiosis of gastric microbiota may promote the development of helicobacter pylori induced gastric malignancies [119]. 

## 6. Concluding Remarks and Future Directions

Since its introduction by George Papanicolaou in 1940’s, the conventional Pap test for the cytological evaluation of cervical cells has emerged as the most extensively utilized cancer screening technique worldwide and has contributed to saving millions of women’s lives. However, despite the tremendous success of the Pap test in cervical cancer screening, this cancer still represents a major public health issue, especially in the developing world, where more than 85% of deaths attributed to cervical cancer occur, hence developing new screening strategies is urgently needed. In this regard, new screening methods are under active investigation. For example, the analysis of biomarkers of virus persistence or cellular transformation is an area of active interest and has potential utility for the screening of other HPV-induced cancers [120,121]. In addition, the use of artificial intelligence algorithms, including machine learning, deep learning and other powerful tools have a tremendous potential for the development of more effective screening strategies given the possibility to combine and decipher massive amount of data from imaging studies, microbiome analysis coupled with clinical patient data [122,123].

A recent Cochrane Systematic Review concluded that there is “high-certainty evidence” that HPV vaccines protect against cervical pre-cancer in adolescent girls and young women aged 15 to 26 and this effect is greater in those who are negative for HPV16/18 or other high risk HPVs at enrolment than those unselected for HPV DNA status [124]. While the use of vaccines for the prevention of HPV infection has the potential to eradicate HPV-induced cancers, worldwide HPV vaccinations has been achieved in less than 2% of females 9–45 years of age [74] and due to the high cost of these vaccines and requirement for special conditions for their preservation, it is expected that HPV-associated malignancies will not significantly decline in regions with limited access to regular screening and vaccination programs, which is also where the incidence rate of cervical cancer is the highest. In addition, the overall impact of vaccination will need decades to be broadly observed since currently available vaccines are not effective to eliminate acquired infections, therefore, in the meantime, other alternatives, such as the development of antiviral agents along with therapeutic vaccines to treat HPV post-infection are urgently needed. In this regard, numerous immunotherapy approaches are currently under evaluation for the treatment of high risk/locally advanced and recurrent/metastatic cervical cancer [125]. Data from phase I and II clinical trials are promising, especially those involving checkpoint inhibitors combined with radiotherapy and chemotherapy, however; the optimal timing for starting immunotherapy is unclear and specific biomarkers predictive of clinical response are missing [126,127,128].

**Figure 2:**
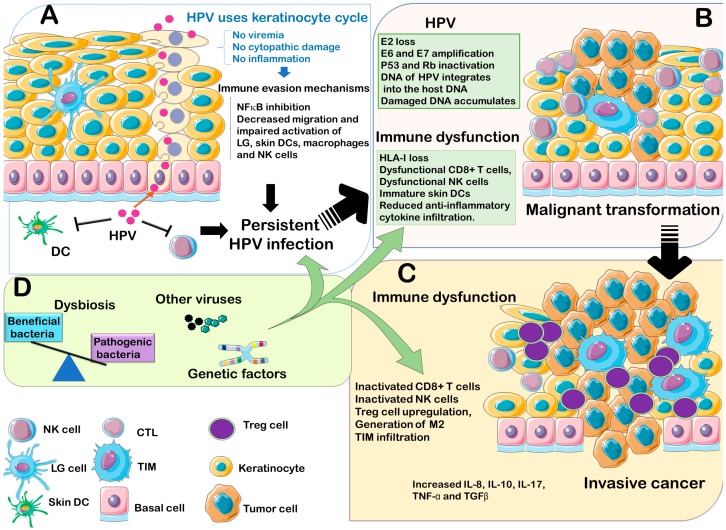
Main factors involved in HPV-induced carcinogenesis. (**A**) Establishing a persistent infection. HPVs have evolved to synchronize their viral cycle to the natural process of keratinocytes differentiation. During this process, the HPV virions are released once keratinocytes complete their differentiation process and therefore, no danger signals are activated and no inflammatory reaction is induced during this cycle. In addition, HPVs manage to avoid immune cells activation by several mechanisms, including inhibition NFκB in keratinocytes and in local immune cells, thus preventing the release of proinflammatory signals. HPVs induce the downregulation of TLR9 expression on keratinocytes, and also impair the local infiltration and activation of antigen presented cells and other components of the innate immune cells, such as skin dendritic cells, macrophages, Langerhans cells and NK cells, ultimately leading to the establishment and maintenance of a persistent infection. (**B**) Malignant transformation. Once a persistent infection has established, the immune system can eventually clear the virus after months or years but in some individuals the infection with high risk HPVs induces malignant transformation. During this process, the viral genome is integrated into the host DNA and is associated with the loss of E2 and the concomitant amplification of E6 and E7 which induces the inactivation of tumor repressors p53 and Rb. In parallel, the immune system fails to detect or eliminate transformed cells leading to tumor formation. (**C**) Tumor progression. During this step, mutations accumulate and immune cells are not only unresponsive to the virus transformed cells but also malignant cells hijack immune system inducing a chronic inflammatory environment with immune cells (Treg, M2 and TIM) that are hostile to anti-tumor immune cells and secrete cytokines and other growth factors that promote the proliferation and invasion of malignant cells. (**D**) Contributing factors. In addition to the viral oncogenic factors and the failure of the immune system to clear HPVs, other factors inherent to the host, such as genetic predisposition, co-infection with other microorganisms, and an altered microbiota may contribute to the carcinogenesis process by affecting the response of the host to the virus infection.

The immune system plays a crucial role in HPV clearance; however, the virus has also developed various immune evasion strategies that can eventually promote persistent infection in the host, which may ultimately result in the development of cancer. A significant increase in circulating NK cells expressing the activator receptors NKG2D, NKp30, NKp46 and ILT2 has been observed following immunization with Gardasil HPV vaccines [129], which may indicate that bolstering NK cells cytotoxicity may contribute to enhance the efficacy of vaccine-induced neutralizing antibodies for the eradication of HPV. In addition to the important role of immune system in the virus elimination, other factors inherent to the host such as the genetic background and the microbiota composition have emerged as additional factors that may influence the susceptibility of a given individual infected with high risk HPV types to develop cancer (Figure 2).

Underscoring the precise role that these factors play in HPV-carcinogenesis, as well as the identification of other relevant factors is crucial for developing new preventive strategies or therapeutic approaches.

Based on their L1 ORF phylogenetic relationships, HPVs are classified into *Alphapapillomavirus, betapapillomavirus, Gammapapillomavirus, Mupapillomavirus* and Nupapillomavirus [130]. This article focused on *Alphapapillomavirus*, which is the most abundant and best characterized HPV genera, however, in recent years, multiple HPV types belonging to lesser characterized genera are being increasingly isolated from human specimens. Among them, *betapapillomavirus* types are linked to the development of skin flat lesions in epidermodysplasia verruciformis patients [131] and serological and epidemiological studies have associated *betapapillomavirus* with non-melanoma skin cancers [132,133,134]. With the development of novel PCR methods and new generation sequencing technologies, novel HPV types are being identified, which highlights the need for their further characterization to elucidate the evolution, anatomical tissue tropisms, the epidemiological associations and medical implications of these viruses.

## Figures and Tables

**Table 1:** Immune escape mechanisms used by human papillomavirus (HPV).

**Effects on Innate Immune System**	**Viral Factor**	**Main Findings/Mechanisms**	**Ref.**
Impairing antiviral activities of keratinocytes.	E6 and E7	E6 and E7 oncogenes repress IFN-κ transcription independently from binding to PDZ proteins. As a result, interferon stimulated genes (ISG), such as IFIT1 and MX1, which have antiviral activities, are inhibited. Similarly, the expression of the pathogen recognition receptors TLR3, RIG-I, and MDA5 in keratinocytes is inhibited. Taken together, our results suggest that HR-HPVs target IFN-κ, which is a master regulator of ISG expression and inducible IFNs in keratinocytes. The expression of proapoptotic genes (TRAIL and XAF1) are also inhibited.	[29]
	E6	Transcriptional repression of E-cadherin by human papillomavirus type 16 E6. Impairs migration and inhibits the maturation of Langerhans cells in the epidermis.	[84]
	E7	HPV decreases CCL20 secretion by keratinocytes by inhibiting CCL20 promoter. E7 oncoviral protein prevents the binding of C/EBP to the CCL20 promoter.	[41]
	E6 and E7	CCL20 production is also affected via downregulation of NFκB signal by E6 and E7	[42]
Impaired antigen presenting cells (APCs)	E6 and E7	HPVs impair the migration, recruitment and localization of dendritic cells to HPV-infected epidermis. HPV impairs the maturation and the function of skin DCs. HR-HPV positive cancer cells, and E6- and E7-expressing cells can inhibit differentiation of monocytes into Langerhans cells.	[35,36]
		Induction of dendritic cells with immunosuppressive activities. Increased expression of programmed death (PD)-1 and its ligand PD-L1 on the surface of dendritic cells.	[85]
Impairing macrophage immune responses	E6	E6 of HPV inhibits the release of monocyte chemotactic protein MCP-1 from infected keratinocytes Downregulate the secretion of MIP1α from infected keratinocytes. As a result, HPV-inhibits macrophage infiltration during acute infection.	[45]
	E6 and E7	During advanced carcinogenesis, HPV tumor infiltrating macrophages (TIM) promote cancer growth and metastasis.	[42]
Impairing NK cells cytotoxicity	E5	E5 induces downregulation of HLA-C and HLA-E on cell surface by sequestering these proteins in intracellular compartments.	[20,51]
	E7	E7 induces the secretion of the immunosuppressive molecule IDO1 from dendritic cells. High levels of IDO1 also found in the serum of patients with HPV-induced CIN 2/3.	[15,55]
		HPV+ tumor cells shed NKG2D-Ls leading to elevated levels of these ligands in the blood of patients with HPV-induced cancers, which ultimately leads to downregulation of NKG2D receptor expression on NK cells thus impairing NKG2D-mediated cytotoxicity.	[53]
Inhibition of NFκB signal pathway	E6 and E7	E7 oncogene, and to a lesser extend E6, strongly reduce NFκB activation and this strongly impairs immune response. As a result, the production of proinflammatory cytokines (IL-1, IL-6 TNF-α, IFN-α and IFN-β) is severely compromised.	[26]
Downregulation of TLR9 expression	E6 and E7	E6 and E7 oncoproteins directly bind to TLR9 promoter and down-regulate TLR9 transcriptional.	[32]
Inhibition of CXCL14	E7	E7 induces hypermethylation of CXCL14 promoter which downregulates CXCL14 expression. CXCL14 deficiency impairs immune cells infiltration to the site of HPV-infection. NK cells, and T cells are especially affected by CXCL14 deficiency.	[56]
		CXCL14 deficiency impairs the differentiation of CD14+ monocytes into Langerhans cells	[59]
		Low levels of CXCL14 may impair macrophage maturation	[60]
Induction of TGF-β1 secretion	E6 and E7	E6 and E7 indirectly interact with the TGF-β1 regulatory element site (GGGGCGG) and activate TGF-β1 promoter. High levels of TGF-β1 in the blood impairs immune cells responses. For example, the expression of activator receptors NKp30 NKp46 and NKG2D on NK cells are severely downregulated resulting in impaired cytotoxicity. High levels of TGF-β1 downregulates MHC class II expression which impairs antigen presenting function by APC.	[61,62]
Induction of IL-10 secretion	E2	E6 and E7 bind to IL-10 promoter thus increasing IL-10 transcription. E2 protein binds to the regulatory region of the human IL-10 gene (-2054 nt) and induces high promoter activity in epithelial cells. High levels of IL-10 impair the cytotoxicity of NK and T cells. IL-10 also promotes TIM infiltration.	[61,86]
		IL-10 stimulates HPV E6 and E7 expression.	[87]
**Effects on Adaptive Immune System**	**Viral Factor**	**Main Findings**	**Ref.**
Impairment of humoral immune response	E7	Induction of a shift from a Th1 response to a Th2 immune response. Upregulation of Th2 cytokines (IL-6, IL-8, and IL-10).	[88]
	E7	CD4^+^ T cells from HPV-associated lesions have impaired production of IL-1β, IL-18, IL2, IFN-γ, and TNF-α	[23]
Promoting immunosuppressive Treg cells	E2, E6, E7	HPV-induced IL-10 and TGF-β1 promote the proliferation of Treg cells. Treg cells accumulate in HPV-transformed tissues. Treg cells suppress Th1 immune responses. Treg cells (CD25/FOXP3 and CD4/TGF-β) secrete immunosuppressive cytokines IL-10 and TGF-β harnessing antiviral and antitumor response of CD8+ CTL and CD4+T cells	[83]
		Activated Treg cells promote the differentiation of monocytes into an immunosuppressive M2-like phenotype	[79]
Impaired cytotoxic T cells activity	E7	E7 directly interacts and block MHC-I heavy chain promoter leading to reduced MHC-I expression on infected keratinocytes and reduces target cell recognition by CD8+ T cells.	[50]
	E5	Downregulation or MHC class II in human keratinocytes leading to impaired APC and poor antigen recognition	[89]
	E5	Upregulation of ganglioside GM1, on the cell surface of HPV-transformed cells leading to cytotoxic T lymphocytes inhibition Induction of PD1 in CD8+ T cells and PDL-1 in DCs and HPV-transformed cells leading to impaired immune surveillance and immune escape.	[68,70,90]
	E7	Microparticles shed from HPVs infected keratinocytes may suppress the cytotoxicity of CD8+ T cells	[71]

## Supplementary Materials

The following are available online at https://www.mdpi.com/2076-2607/7/7/199/s1. Supplementary Table S1. Genetic polymorphisms associated with HPV-induced cancers susceptibility.
