# Supplementary material for: The Host-Microbe Interplay in Human Papillomavirus-Induced Carcinogenesis
Source: Microorganisms. 2019 Jul 13;7(7):199. doi: 10.3390/microorganisms7070199 (PMC6680694; doi:10.3390/microorganisms7070199)
Supplement: Supplementary file 1 [file microorganisms-07-00199-s001.pdf]

Sup. Table S1. Genetic polymorphisms associated with HPV-induced cancers susceptibility

Type of study (GWAS or target SNP)	Type of cancer/ N0 of individuals/ Ethnicity	Main findings	Ref.
Target SNP -318 C/T, +49 A/G and CT60 A/G SNP in CTL-4 gene	Cases: women with cervical squamous cell carcinoma (n=144) and Controls (n= 378) Taiwanese	The -318 C/T variant in the promoter region of the CTLA-4 gene is associated with HPV-16-associated CSCC	[1]
Target SNP Inflammasome genes (NLRP1, NLRP3, NLRP6, CARD8, IL1B, IL18, TNFAIP3)	HPV+ case (n=246) Controls (n =310) Brazilians	SNPs rs1143643 (L1B gene) was associated with protection against HPV infection. SNPs rs11651270 and rs10754558 (NLRP1) were associated with protection against HPV persistence and/or oncogenesis. SNP rs10754558 (NLRP3) was associated with significantly lower risk to be infected with a high-risk HPV	[2]
Target SNP SNPs rs25164488, rs3117027 and rs9272143	Cases: cervical cancer (n=790) and Controls (n=717) from Algeria, Morocco, India and Thailand	SNP rs2844511 (CHR6) and cervix cancer risk. Borderline associations between rs2665390 (TIPARP) and rs13117307 (EXOC1) with cervical cancer risk. Also confirmed the association between rs2844511 and cervical cancer risk as previously reported in a Swedish population.	[3]
SNP array genotyped 92 SNPs from 49 candidate immune response and DNA repair genes	Women with CIN3 or cancer (n=469), Women with persistent HPV infections (n=390), and Controls (n=452) Costa Rica	SNP G501S (FANCA gene) was associated with increased risk of CIN3 or cancer. The FANCA haplotype that included G501S also conferred increased risk of CIN3 or cancer. A SNP in the innate immune gene IRF3 (S427T) was associated with increased risk for HPV persistence	[4]
Target SNP rs1049174 NKG2D gene	153 women with HPV+cervical cancer	LNK was significantly associated with increased cancer susceptibility to HPV+ cancers	[5]

	123 patients with HPV+ anogenital cancers<br><br>Cervical and anogenitalcancers<br><br>Vietnamese	LNK was significantly associated with lower NKG2D expression in NK cells and lower cytotoxicity	
Target SNP<br>p73 cytosine thymine	Cases:<br>HSIL (n=38)<br>Invasive cervical cancer (n=141)<br>Controls (n=176) from Portugal	Twofold increased susceptibility to the development of HSIL in women carrying the p73 AT allele (OR=2.39; p=0.022) which was specially evident in women with high parity.	[6]
Array genotype<br>SNPs at the HLA class II *DQB1* typed using a linear array of immobilized sequence-specific oligonucleotide probes	Cases: cervical cancer (n=1306)<br><br>Controls (n=288)<br><br>Sweden	*DQB1*was strongly associated; alleles **0301*, **0402* and **0602*increased cancer susceptibility, whereas **0501* and **0603*decreased susceptibility.	[7]
HLA-DRB1*1501 and DQB1*0602 by PCR-SSP analysis	Cases invasive cervical cancer (n=287) (192 Chinese Uighurs and 95 Hans)<br>Healthy controls (n=312 Chinese 218 Uighurs and 94 Hans)	The HLA-DQB1*0602 allele frequency was significantly lower among Uighur women with invasive cervical cancer. Similar tendencies were observed for DQB1*0602 with HPV16-positive invasive cervical cancer.	[8]
HLA-DRB1*13 allele	86 women with CIN1 Low-Grade Cervical Intraepithelial Neoplasia<br><br>France	HLA-DRB1*13 allele and HPV16/18 negative status were independently associated with an increased probability for regression	[9]

HLA-DRB1*1302 allele	Cases: CIN1 (n=505)<br>CIN2/3 (n=96)<br>Invasive cervical cancer (n=311)<br>Controls (n=341)<br>Japanese	Protective effect of HLA-DRB1*1302 allele against progression from CIN 1 to CIN2/3	[10]
Array genotype including 205 SNPs in and around 32 candidate gene regions	Cases<br>Cervical cancer (n=876)<br>Vulvar cancer (n=517)<br><br>Controls (n=1100)<br>United States	The TNF region was significantly associated with the risks of cervical cancer and vulvar cancer.<br>The allele A of the SNP rs2239704 LTA gene associated with increased risk of cervical cancer and of vulvar cancer.<br><br>SNP rs2239704 in TLR gene was no associated with these cancers	[11]
Systematic review and m*eta-analysis* to establish the associations between cancers and *LTA* variants (rs1041981, rs2239704, rs2229094 and rs746868)	A total of 30 case-control studies involving 58,649 participants	rs1041981 increased the risk of several types of cancer, such as adenocarcinoma, squamous carcinoma, hematological malignancy in Asians, Europeans.	[12]
Target SNP rs3087404 and rs2029167 in SMUG1 gene	Cases<br>cervical squamous cell carcinoma (n=400)<br><br>CIN III (n=400)<br><br>Chinese	The homozygous GG of rs3087404 and rs2029167 had a significantly increased risk of CIN III and cervical squamous cell carcinoma<br><br>Individuals with G allele or G carrier at rs3087404 were at higher risk for cervical squamous cell carcinoma, and at rs2029167 were at higher risk for CIN III	[13]
Target Ser326Cys polymorphism in hOGG1(	Cases CIN grade III (n=400)	The genotype hOGG1 Cys326Cys (GG) was associated with increased risk of CIN	[14]

human 8-oxoguanine glycosylase 1)	Cervical squamous cell carcinoma (n=400)<br>Chinese	III and cervical squamous cell carcinoma and also in HR-HPV infected.	
Target SNP rs11637235 of the DUT gene	Cases<br>Cervical squamous cell carcinoma (n=400)<br>CIN<br>(n=400)<br>Chinese	GG genotype of rs3784619 and the TT genotype of rs11637235 in the DUT gene significantly increased the risk of CIN III and cervical squamous cell carcinoma<br>The TT genotype of rs11637235 was enriched in the HR-HPV–positive cases	[15]
Target 3DS1 and 2DS1 in KIR gene	Cases:<br>RRP patients (n=66)<br>United States<br>(Caucasians, African Americans, Hispanics)	Individuals lacking activating KIR genes 3DS1 and 2DS1 are more likely to develop a more severe form of RRP (caused by HPV-6/11) than those harboring these receptors	[16]
Systematic review and m*eta-analysis*	Meta –analysis<br><br>Cervical cancer<br><br>Indian	rs1048943 A>G, in exon 7 of CYP1A1 to be associated with cervical cancer	[17]
Target SNP rs1982073 of TGF-β1 gene	Cases: squamous cell carcinoma of the oropharynx (n=200)<br>HPV16+ patients (n = 147) HPV16- patients (n = 53)<br>United States	*Patients with rs1982073 CT/CC genotypes* were significantly associated with HPV16-positive tumor status among patients with squamous cell carcinoma compared with TT genotype	[18]
GWAS	Cervical cancer<br>1075 Cases and 4014 controls<br>Sweden	Three loci: rs2516448 in MICA gene; rs9272143 between HLA-DRB1 and HLA-DQA1 genes and rs3117027 at HLA-DPB2 gene were associated with susceptibility to cervical cancer.<br><br>The study also confirmed previously reported associations of B*0702 and DRB1*1501-DQB1*0602 with susceptibility to and DRB1*1301-DQA1*0103-	[19]

		DQB1*0603 with protection against cervical cancer	
SNP array including 7140 tag SNPs from 305 candidate genes/regions using custom-designed iSelect Infinium assay	Cases 1 (n=416), (CIN3)/cancer<br><br>Cases 2 (n=356), Persistent HPV women (median: 25 months),<br><br>Control (n=425) random controls<br><br>Costa Rica	SNPs rs12302655 (OAS3), rs4737999 (SULF1), rs3784621 (DUT), and rs2894054 (GTF2H4) were associated with HPV persistence<br><br>SNPs rs11177074 (IFNG) and rs9893818 (EVER1/EVER2) were associated with progression to CIN3/cancer.	[20]
Target SNP<br><br>rs2910164(G>C) on the passenger strand of the precursor of miR-146a	Chinese Cervical cancer cases (n=447) and Controls (n=443)	Subjects carrying GG homozygote had a 1.496-fold increased risk than those carrying CG/CC genotypes.<br><br>Carriers of GG genotype had lower miR-146a expression level compared with the carriers of CC genotype.	[21]
Target SNP<br>TP53, rs1042522<br>*MDM2* (SNP309)<br>and *NQO1* (SNP609, SNP465)	Patients with cervical cancer (n=577) and their biological parents and/or siblings	No association between *MDM2* SNP309 or *NQO1* SNP465 and cervical cancer. *TP53* codon 72 and *NQO1* SNP609 associate with higher risk of cervical cancer especially in women infected with HPVs 16–and/or 18.	[22]

Foot notes:

GWAS study: genome-wide association study; SNP: single nucleotide polymorphism

REFERENCES

[1] T.H. Su, T.Y. Chang, Y.J. Lee, C.K. Chen, H.F. Liu, C.C. Chu, M. Lin, P.T. Wang, W.C. Huang, T.C. Chen, Y.C. Yang, CTLA-4 gene and susceptibility to human papillomavirus-16-associated cervical squamous cell carcinoma in Taiwanese women, Carcinogenesis, 28 (2007) 1237-1240.

[2] A. Pontillo, L.A. Brandão, R.L. Guimarães, L. Segat, E. Athanasakis, S. Crovella, A 3'UTR SNP in NLRP3 gene is associated with susceptibility to HIV-1 infection, J Acquir Immune Defic Syndr, 54 (2010) 236-240.
[3] J. McKay, V. Tenet, S. Franceschi, A. Chabrier, T. Gheit, V. Gaborieau, S. Chopin, P.H. Avogbe, M. Tommasino, M. Ainouze, U. Hasan, S. Vaccarella, Immuno-related polymorphisms and cervical cancer risk: The IARC multicentric case-control study, PLoS One, 12 (2017) e0177775.
[4] S.S. Wang, M.C. Bratti, A.C. Rodríguez, R. Herrero, R.D. Burk, C. Porras, P. González, M.E. Sherman, S. Wacholder, Z.E. Lan, M. Schiffman, S.J. Chanock, A. Hildesheim, Common variants in immune and DNA repair genes and risk for human papillomavirus persistence and progression to cervical cancer, J Infect Dis, 199 (2009) 20-30.
[5] J.L. Espinoza, V.H. Nguyen, H. Ichimura, T.T. Pham, C.H. Nguyen, T.V. Pham, M.I. Elbadry, K. Yoshioka, J. Tanaka, L.Q. Trung, A. Takami, S. Nakao, A functional polymorphism in the NKG2D gene modulates NK-cell cytotoxicity and is associated with susceptibility to Human Papilloma Virus-related cancers, Sci Rep, 6 (2016) 39231.
[6] R. Craveiro, I. Bravo, R. Catarino, A.L. Teixeira, H. Sousa, D. Pereira, H. Pereira, R. Medeiros, The role of p73 G4C14-to-A4T14 polymorphism in the susceptibility to cervical cancer, DNA Cell Biol, 31 (2012) 224-229.
[7] E.L. Ivansson, J.J. Magnusson, P.K. Magnusson, H.A. Erlich, U.B. Gyllensten, MHC loci affecting cervical cancer risk: distinguishing the effects of HLA-DQB1 and non-HLA genes TNF, LTA, TAP1 and TAP2, Genes Immun, 9 (2008) 613-623.
[8] J.M. Hu, Q. Sun, L. Li, C.X. Liu, Y.Z. Chen, H. Zou, L.J. Pang, J. Zhao, L. Yang, Y.W. Cao, X.B. Cui, Y. Qi, W.H. Liang, W.J. Zhang, F. Li, Human leukocyte antigen-DRB1*1501 and DQB1*0602 alleles are cervical cancer protective factors among Uighur and Han people in Xinjiang, China, Int J Clin Exp Pathol, 7 (2014) 6165-6171.
[9] X. Sastre-Garau, I. Cartier, N. Jourdan-Da Silva, P. De Crémoux, V. Lepage, D. Charron, Regression of low-grade cervical intraepithelial neoplasia in patients with HLA-DRB1*13 genotype, Obstet Gynecol, 104 (2004) 751-755.
[10] K. Matsumoto, H. Maeda, A. Oki, N. Takatsuka, T. Yasugi, R. Furuta, R. Hirata, A. Mitsuhashi, K. Kawana, T. Fujii, T. Iwata, Y. Hirai, M. Yokoyama, N. Yaegashi, Y. Watanabe, Y. Nagai, H. Yoshikawa, J.H.a.C.C.J.S. Group, Human leukocyte antigen class II DRB1*1302 allele protects against cervical cancer: At which step of multistage carcinogenesis?, Cancer Sci, 106 (2015) 1448-1454.
[11] C. Bodelon, M.M. Madeleine, L.G. Johnson, Q. Du, D.A. Galloway, M. Malkki, E.W. Petersdorf, S.M. Schwartz, Genetic variation in the TLR and NF-κB pathways and cervical and vulvar cancer risk: a population-based case-control study, Int J Cancer, 134 (2014) 437-444.
[12] Y. Huang, X. Yu, L. Wang, S. Zhou, J. Sun, N. Feng, S. Nie, J. Wu, F. Gao, B. Fei, J. Wang, Z. Lin, X. Li, L. Xu, X. Gao, M. Ye, S. Duan, Four genetic polymorphisms of lymphotoxin-alpha gene and cancer risk: a systematic review and meta-analysis, PLoS One, 8 (2013) e82519.
[13] F. Ye, H. Wang, J. Liu, Q. Cheng, X. Chen, H. Chen, Association of SMUG1 SNPs in Intron Region and Linkage Disequilibrium with Occurrence of Cervical Carcinoma and HPV Infection in Chinese Population, J Cancer, 10 (2019) 238-248.
[14] H. Chen, H. Wang, J. Liu, Q. Cheng, X. Chen, F. Ye, Association of Base Excision Repair Gene hOGG1 Ser326Cys Polymorphism with Susceptibility to Cervical Squamous Cell Carcinoma and High-Risk Human Papilloma Virus Infection in a Chinese Population, Genet Test Mol Biomarkers, 23 (2019) 138-144.
[15] F. Ye, H. Wang, J. Liu, Q. Cheng, X. Chen, H. Chen, Genetic variants of the dUTPase-encoding gene DUT increase HR-HPV infection rate and cervical squamous cell carcinoma risk, Sci Rep, 9 (2019) 513.
[16] V.R. Bonagura, Z. Du, E. Ashouri, L. Luo, L.J. Hatam, J.A. DeVoti, D.W. Rosenthal, B.M. Steinberg, A.L. Abramson, D.W. Gjertson, E.F. Reed, R. Rajalingam, Activating killer cell immunoglobulin-like receptors 3DS1 and 2DS1 protect against developing the severe form of recurrent respiratory papillomatosis, Hum Immunol, 71 (2010) 212-219.
[17] D. Sengupta, U. Guha, S. Mitra, S. Ghosh, S. Bhattacharjee, M. Sengupta, Meta-Analysis of Polymorphic Variants Conferring Genetic Risk to Cervical Cancer in Indian Women Supports CYP1A1
as an Important Associated Locus, Asian Pac J Cancer Prev, 19 (2018) 2071-2081.
[18] X. Guan, E.M. Sturgis, D. Lei, Z. Liu, K.R. Dahlstrom, Q. Wei, G. Li, Association of TGF-beta1 genetic variants with HPV16-positive oropharyngeal cancer, Clin Cancer Res, 16 (2010) 1416-1422.
[19] D. Chen, I. Juko-Pecirep, J. Hammer, E. Ivansson, S. Enroth, I. Gustavsson, L. Feuk, P.K. Magnusson, J.D. McKay, E. Wilander, U. Gyllensten, Genome-wide association study of susceptibility loci for cervical cancer, J Natl Cancer Inst, 105 (2013) 624-633.
[20] S.S. Wang, P. Gonzalez, K. Yu, C. Porras, Q. Li, M. Safaeian, A.C. Rodriguez, M.E. Sherman, C. Bratti, M. Schiffman, S. Wacholder, R.D. Burk, R. Herrero, S.J. Chanock, A. Hildesheim, Common genetic variants and risk for HPV persistence and progression to cervical cancer, PLoS One, 5 (2010) e8667.

[21] C. Yue, M. Wang, B. Ding, W. Wang, S. Fu, D. Zhou, Z. Zhang, S. Han, Polymorphism of the pre-miR-146a is associated with risk of cervical cancer in a Chinese population, Gynecol Oncol, 122 (2011) 33-37.
[22] X. Hu, Z. Zhang, D. Ma, P.C. Huettner, L.S. Massad, L. Nguyen, I. Borecki, J.S. Rader, TP53, MDM2, NQO1, and susceptibility to cervical cancer, Cancer Epidemiol Biomarkers Prev, 19 (2010) 755-761.
